# MALDI-MSI-LC-MS/MS Workflow for Single-Section Single Step Combined Proteomics and Quantitative Lipidomics

**DOI:** 10.1021/acs.analchem.3c05850

**Published:** 2024-03-04

**Authors:** Tim F.E. Hendriks, Kasper K. Krestensen, Ronny Mohren, Michiel Vandenbosch, Steven De Vleeschouwer, Ron M.A. Heeren, Eva Cuypers

**Affiliations:** † The Maastricht MultiModal Molecular Imaging (M4I) institute, Division of Imaging Mass Spectrometry (IMS), Maastricht University, 6229 ER Maastricht, The Netherlands; ‡ Department of Neurosurgery, Laboratory for Experimental Neurosurgery and Neuroanatomy, UZ Leuven, KU Leuven, 3000 Leuven, Belgium

## Abstract

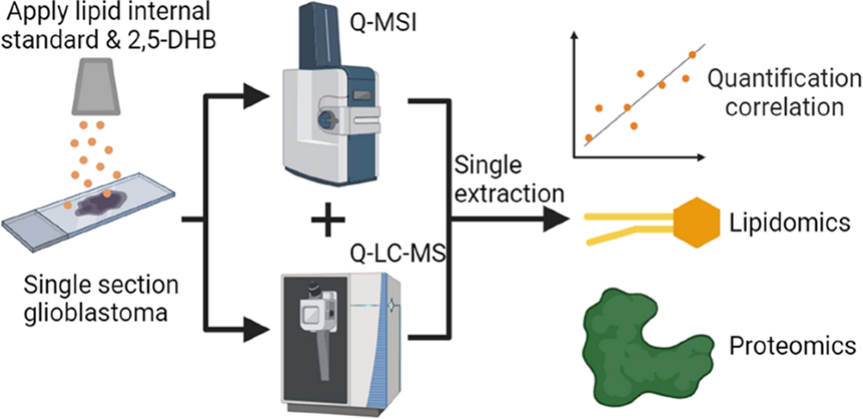

We introduce a novel approach for comprehensive molecular profiling in biological samples. Our single-section methodology combines quantitative mass spectrometry imaging (Q-MSI) and a single step extraction protocol enabling lipidomic and proteomic liquid chromatography tandem mass spectrometry (LC-MS/MS) analysis on the same tissue area. The integration of spatially correlated lipidomic and proteomic data on a single tissue section allows for a comprehensive interpretation of the molecular landscape. Comparing Q-MSI and Q-LC-MS/MS quantification results sheds new light on the effect of MSI and related sample preparation. Performing MSI before Q-LC-MS on the same tissue section led to fewer protein identifications and a lower correlation between lipid quantification results. Also, the critical role and influence of internal standards in Q-MSI for accurate quantification is highlighted. Testing various slide types and the evaluation of different workflows for single-section spatial multiomics analysis emphasized the need for critical evaluation of Q-MSI data. These findings highlight the necessity for robust quantification methods comparable to current gold-standard LC-MS/MS techniques. The spatial information from MSI allowed region-specific insights within heterogeneous tissues, as demonstrated for glioblastoma multiforme. Additionally, our workflow demonstrated the efficiency of a single step extraction for lipidomic and proteomic analyses on the same tissue area, enabling the examination of significantly altered proteins and lipids within distinct regions of a single section. The integration of these insights into a lipid–protein interaction network expands the biological information attainable from a tissue section, highlighting the potential of this comprehensive approach for advancing spatial multiomics research.

## Introduction

A single-section approach for quantitative (Q) mass spectrometry imaging (MSI) followed by liquid chromatography tandem mass spectrometry (LC-MS/MS) proteomics and lipidomics holds significant importance in research that focuses on the in-depth understanding of the spatial and molecular dynamics within a biological sample.^1^ This integrated strategy on a single tissue section allows for direct spatial correlation between lipidomic and proteomic data and MSI results, enabling a comprehensive visualization and interpretation of the molecular landscape.^2^ Moreover, the integration of data from different omics platforms in the same section facilitates a more nuanced exploration of the interaction between up- and downregulated lipid and protein pathways.^3^ The challenge so far has been that proteomic and lipidomic LC-MS-based analysis following laser capture microdissection needed to be applied to either consecutive sections or separate areas from the same tissue section. Here, we developed an approach that allows both proteomic and lipidomic analyses on the exact same selected group of cells excised from a single tissue section.

A comparison between Q-MSI and Q-LC-MS/MS is crucial in the optimization of analytical approaches for comprehensive molecular profiling.^4^ Q-MSI excels in providing quantitative spatial information and allows for the visualization of molecular distributions.^5^ On the other hand, Q-LC-MS/MS offers high sensitivity and specificity, which allows for in-depth characterization of molecular species but lacks the spatial context crucial for understanding localized molecular changes within a tissue. Integrating both approaches on a single tissue section provides a synergy that leverages the spatial insights from MSI and the molecular specificity from LC-MS/MS.^6^ This facilitates a more comprehensive analysis of the microenvironment for both diagnostic and therapeutic advancements.

The importance of spraying an internal standard preceding Q-MSI cannot be understated. These known reference compounds, sprayed onto the sample prior to analysis, play a crucial role in the accuracy and reliability of quantitative measurements.^7^ Internal standards help correct for variations in ionization efficiency and matrix effects across different regions of the tissue, providing more accurate quantification.^8^

Integrating a single-section approach combining Q-MSI and Q-LC-MS would be beneficial in disease-related research in which sample amount is limited, such as patient tissue. One example is glioblastoma multiforme (GBM), an aggressive form of brain cancer, exhibiting complex molecular heterogeneity.^9^ Only a limited amount of material after diagnostic analysis is left for research for these diseases. As such, a comprehensive analysis is essential for unraveling its complex biology.^10^ A combined analytical strategy on one single tissue section not only preserves precious samples, crucial in the context of limited brain tissue availability, but also allows for an in-depth examination of the highly heterogeneous spatial distribution of lipids and proteins within the tumor microenvironment.^2^ Even though consecutive sections can provide an understanding of the surrounding environment, section-to-section variability is expected.^11^ Understanding the molecular details of GBM is essential for advancing therapeutic strategies, and the integrative power of combining Q-MSI, lipidomics, and proteomics into a single section holds promise for discovering novel biomarkers and potential therapeutic targets in this disease.^12^

**Figure 1:**
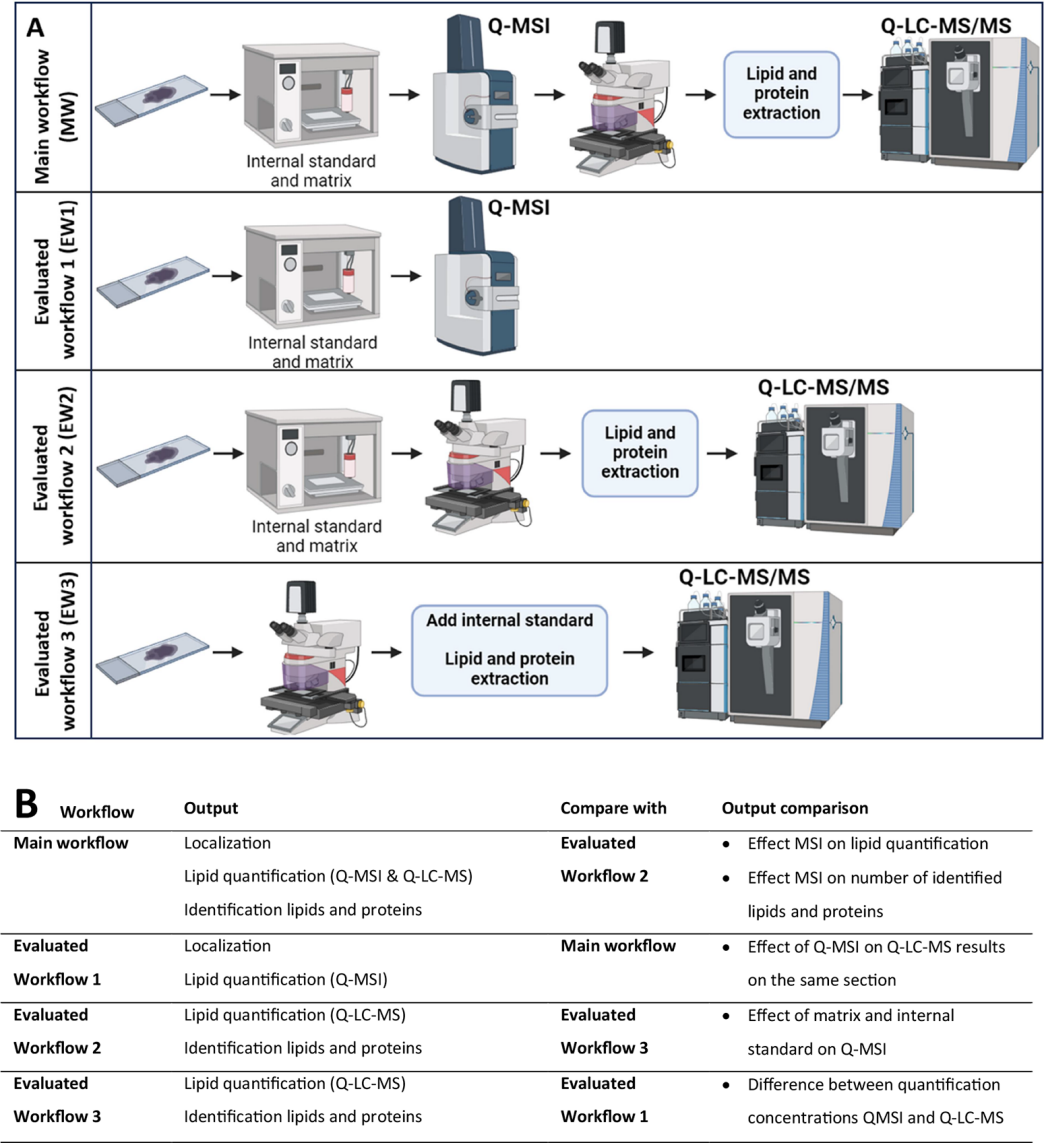
(A) Illustration of the main and evaluated workflows. (B) Table that shows the output of the workflows and the output of the comparison between workflows.

Here, we propose a main workflow (MW) (Figure 1) using Q-MSI, image-guided segmentation and dissection, and a single step extraction for proteomics and quantitative lipidomics LC-MS/MS analysis on a single GBM tissue section. Briefly, MSI-based annotated regions of interest (ROI) are dissected using laser capture microdissection (LMD) after matrix-assisted laser desorption ionization (MALDI) with post-ionization (MALDI-2)-MSI. The use of a post-ionization laser in MALDI-2 provides increased ion yields and allows for a significantly expanded number of lipid classes that can be studied and quantified.^7,13^ A single step two-phase extraction was applied to the dissected tissue to enable proteomic and lipidomic LC-MS/MS analysis on the same tissue area after MSI. Several other evaluated workflows (EW) were compared and contrasted with the main workflow to assess the quantitative and qualitative results. Comparing Q-MSI and Q-LC-MS/MS data allowed for assessment of the quality of MSI-quantification. The influence of different slide types, including polyethylene napthalate (PEN)-membrane slides, IntelliSlides, and indium tin oxide (ITO)-slides on the quantification and identification, was also assessed. Afterward, we performed a joint-pathway analysis for proteins and lipids using the significantly altered compounds found via the proposed workflow.

## Materials and Methods

### Chemicals

2,5-dihydroxybenzoic acid (2,5-DHB), ammonium bicarbonate (ABC), ammonium formate (AF), dithiothreitol (DTT), Entellan mounting medium, ethanol (EtOH), eosin-Y, formic acid (FA), Gill’s hematoxylin, iodoacetamide (IAM), methyl-tert butyl ether (MTBE), and xylene were purchased from Sigma-Aldrich (The Netherlands). 2-propanol (IPA), acetonitrile (ACN), methanol (MeOH), trifluoroacetic acid (TFA), and water (H2O) were purchased from BioSolve (The Netherlands). The RapiGest (RG) surfactant was purchased from Waters (USA). Enzyme mix (trypsin/Lys-C) was purchased from Promega (United States). PEN-membrane slides and Pierce FlexMix Calibration Solution (A39239) were purchased from Thermo Fisher (The Netherlands). ITO slides were purchased from Delta Technologies (USA). IntelliSlides were purchased from Bruker Daltonik GmbH (Germany). MSI SPLASH mix (330841) was purchased from Avanti Polar Lipids (USA) (detailed composition is given in Table S1). All reagents were of LC-MS grade.

### Sample Preparation

Human GBM was sectioned at 10 μm thickness using a cryotome (CM1850, Leica Biosystems), and consecutive sections were thaw-mounted on ITO, PEN-membrane slides, and IntelliSlides. MSI SPLASH mix was sprayed when indicated as an internal standard on all slide types. The internal standard was prepared by diluting the stock solution 10 times with LC-MS grade-MeOH prior to deposition. The spraying parameters for the internal standard and 2,5-DHB matrix are provided in the Supporting Information.

### MALDI-2-TOF Mass Spectrometry Imaging

MALDI-2-MSI analysis was performed on a MALDI-2-timsTOF flex instrument (Bruker Daltonics GmbH, Germany). Data were obtained in positive ion mode with a mass range of *m*/*z* 350–1000 and a pixel size of 30 × 30 μm. The laser frequency was set to 1 kHz for the MALDI-1 and MALDI-2 lasers, and 100 shots were accumulated at each pixel. The MALDI-1 laser consisted of a Nd:YAG 355 nm SmartBeam 3D laser (Ekspla, Lithuania). The MALDI-2 effect was obtained by a perpendicular 266 nm NL 204-1k-FH laser (NL204-1K-FH, Ekspla, Lithuania) with a pulse delay of 10 μs. Before the imaging experiments, time-of-flight calibration was performed by using red phosphorus.

### Image Visualization and Segmentation

MALDI-MSI data were visualized in LipostarMSI v2.0.1b (Molecular Horizon), and peak picking was performed with a minimum signal-noise-ratio of 1 and a noise window size of 0.1 Da, and peaks under 1% of the base peak were discarded. Savitsky–Golay smoothing settings were window size 7 points; degree 2 and iterations 1. A signal-to-noise ratio of 3 was used for data processing, quantification, and identification. The whole tissue was segmented via bisecting K-means clustering with a medium denoising strength using a biomarker list of all selected peaks between *m*/*z* 600 and 1000 normalized by the total ion count (TIC). The segmentation was performed until two different subpopulations were visualized that represented the tumor and necrotic regions of the GBM sections. These subpopulations were coregistered with the annotated hematoxylin and eosin (H&E)-stained images (see protocol in the Supporting Information). In each subpopulation, the coordinates of a region of approximately 1 mm^2^ were generated and exported to the LMD microscope for laser capture microdissection (see the protocol in the Supporting Information).

### Single Step Extraction for Lipidomics and Proteomics

Lipids and proteins were extracted as follows: 400 μL of MTBE was added to the LMD dissected material in MeOH, vortexed for 1 min, and placed in a thermoshaker at 20 °C and 950 rpm for 1 h. Next, 100 μL of water was added and vortexed for 1 min, followed by centrifuging at 1000 g for 10 min in an Eppendorf centrifuge 5353 (Eppendorf).

The lipid fraction was separated by the transfer of the upper organic fraction to a new 2 mL tube. Here, re-extraction of lipids was performed by adding 300 μL of MTBE, 40 μL of MeOH, and 30 μL of water to the bottom aqueous fraction and vortexed for 1 min. The sample was centrifuged again for 10 min at 1000g. The upper organic fractions were combined per sample. The organic fractions were completely dried in a vacuum centrifuge (Heto Lab), reconstituted in 50 μL 1:1 ACN:IPA, and used for LC-MS/MS (Vanquish UHPLC, HypersilGOLD 100 × 2.1 mm, and Orbitrap Exploris480, Thermo Fisher) lipidomic analysis based on a generic lipidomics protocol.^14^ The aqueous bottom fraction was vacuum-dried prior to proteomic analysis.

The dried protein fraction was dissolved in 20 μL of 50 mM ABC buffer for subsequent proteome analysis. A volume of 2.2 μL of 0.1% Rapigest, to help with protein solubility, was added to the sample solution and incubated at 21 °C for 10 min at 800 rpm. Next, to break the disulfide bonds, 1.3 μL of 10 mM DTT was added to the solution and incubated at 56 °C for 40 min at 800 rpm. To prevent the reformation of disulfide bonds, the cysteines were alkylated by adding 1.4 μL of 20 mM IAM and incubated at room temperature for 30 min. Afterward, 1.4 μL of 10 mM DTT was added to the mixture and incubated at room temperature for 10 min at 800 rpm. Then, 1 μL of 0.5 μg/μL trypsin was added for an overnight digestion at 37 °C and 800 rpm. After overnight digestion 0.3 μL trypsin and 115 μL ACN were added to the mixture for a final digestion at 37 °C for 3 h at 800 rpm. To terminate the reaction, 6 μL of 10% TFA was added and incubated for 45 min at 37 °C and 800 rpm. Samples were then centrifuged for 15 min at 4 °C at 15.000 g. The supernatant was transferred to a new vial and concentrated in a final volume of 30 μL.

### Lipidomics Acquisition and Identification

The lipid identification was performed on an Orbitrap Exploris480 mass spectrometer (Thermo Fisher Scientific, USA) running in a data-dependent acquisition (DDA) positive mode. Here, MS1 data of *m*/*z* 200–1450 were acquired at a mass resolution of 60,000 with an injection time of 65 ms. In parallel, MS2 data were acquired in the ion trap with collision-induced dissociation (CID) using an isolation window of 1.7 Da and a mass resolution of 30,000. The lipid species in LC-MS were subsequently assigned using MS1 and MS2 spectra acquired from DDA measurements in Lipostar2 version 2.1.2. Lipid identifications for MALDI-MSI were assigned by linking MS1 precursor ions found in the MALDI-MSI measurements to the MS1 + MS2 *m*/*z* values found in the LC-MS/MS measurements. The combination of MS1 (MALDI) and MS2 (LC-MS/MS) was used for identification in LipostarMSI v2.0.1, both using the LIPID MAPS database (3- and 4-star rating, Molecular Horizon, Bettona, PG, Italy).^15^

### Proteomics Acquisition and Identification

Peptide separation was achieved on a Thermo Scientific (Dionex) Ultimate 3000 Rapid Separation UHPLC system with a Thermo Scientific Acclaim PepMap C18 analytical column (150 × 0.75 mm, 3 μm). Peptide samples were desalted on an online installed C18 trapping column. After desalting, peptides were chromatically separated on the analytical column with a 110 min gradient from 4 to 32% ACN with 0.1% FA at 300 nL/min flow rate. The UHPLC system was coupled to a Q Exactive HF mass spectrometer (Thermo Scientific). DDA settings were as follows: MS1 scans between 250 and 1250 *m*/*z* at a resolution of 120000, followed by MS2 scans of the top 15 most intense ions at a resolution of 15000.

Proteomic LC-MS data used for protein identification were analyzed using Proteome Discoverer 3.0 (Thermo Scientific) using the protein database *Homo sapiens* (Uniprot taxonomy ID 9606). The following settings were used for the protein database search: trypsin was used as the enzyme with a maximum of two missed cleavage sites and a minimum peptide length of six amino acids. The mass window for the precursors was set at 350–5000 Da. The mass tolerance of the precursor and fragment were 10 ppm and 0.02 Da, respectively. Acetylation on the n-terminus and methionine oxidation were used as dynamic modifications, and carbamidomethylation was used as static modifications. A strict false discovery rate (FDR) of 0.01 was used to estimate the confidence of the identification. Accession numbers of the proteins were used to assess the protein-encoding gene names via the UniProtKB database.

### Lipid Quantification (Q-) with MSI and LC-MS/MS

The MALDI-2-MSI data were analyzed in SCiLS Lab version 2024a (Bruker Daltonic, Germany). To analyze the Q-LC-MS/MS data, Lipostar v2.1.2 (Molecular Discovery, United Kingdom) was used with a signal filtering threshold of 10,000, and peaks below 1% of the base peak were removed. Q-MSI and Q-LC-MS-based lipid quantification is calculated by determining the lipid peak areas and their respective internal standard of the same lipid species in an ROI. The concentration of the analyte of interest was calculated by multiplying the peak area of the sample with the concentration of the corresponding internal standard. This value was then divided by the peak area of the internal standard. Per lipid, the concentration of their adduct was determined by the adduct of the internal standard, meaning that the [M + H]^+^ concentration of a lipid was determined by the [M + H]^+^ adduct of the internal standard. The concentration of each lipid was determined by summing the concentrations found for the [M + H]^+^, [M + Na]^+^ and [M + K_39_]^+^ adducts.

### Comparison between Generated Workflows

In this study, we established one main workflow (MW). The MW consists of spraying an internal standard and matrix on a single GBM section. Q-MSI is followed by MSI-guided segmentation and LMD. With the LMD dissected material, a single extraction is performed for both lipidomics and proteomics analysis via LC-MS/MS. Three other workflows that are visualized in Figure 1 were used to evaluate the effect of the different process steps in the main workflow. Evaluated workflow 1 (EW1) consists of a previously described protocol that allows for Q-MSI.^7^ In evaluated workflow 2 (EW2), internal standard and matrix were sprayed on a GBM section, and LMD and a single extraction for lipidomic and proteomic LC-MS/MS analysis were performed. The third evaluated workflow (EW3) started by performing LMD without MSI on a GBM section, adding the internal standard before lipidomic and proteomic extraction, followed by LC-MS/MS analysis.

The qualitative and quantitative results from the evaluated workflows were compared to each other and the MW to understand the effect of each workflow element. The MW output enables localization, lipid quantification, and lipid and protein identification (via Q-MSI and (Q-)LC-MS/MS) on a single tissue area. EW1 allows for only localization and lipid quantification via Q-MSI and misses the identification properties for proteins and lipids as present in the MW. EW2 and EW3 only allow for lipid quantification and lipid and protein identification via (Q-)LC-MS/MS and miss the spatial information gained via MSI. When MW and EW1 are compared, the effect of performing MSI on the same section is evaluated. A comparison between MW and EW2 enables the visualization of the effect of MSI on lipid quantification, as well as lipid and protein identification results. The effect of different internal standard and matrix deposition methods was evaluated. In EW2, the internal standard and matrix are sprayed on the tissue prior to dissection, whereas in EW3, internal standard is applied via pipetting to the extract after dissection. A comparison between EW2 and EW3 reveals the effect of matrix and internal standard addition. The comparison between EW1 and EW3 targets a comparison of the quantitative results of MSI and LC-MS. A summary of the output and the comparison of the workflows is depicted in Figure 1.

### Metabolite–Gene Network Analysis

Metaboanalyst 5.0 was used to perform a joint-pathway analysis to identify the biological pathways that differ between the two different regions found in GBM data, conducted from a single section and acquired and processed via the MW. These pathways were based on the up- and downregulated protein-encoding gene names and lipids found when comparing tumor with the necrotic regions in the proteomics and lipidomics data. Proteins and lipids were considered significantly altered when a fold change of 1.5 occurred (log2 ≥ 0.58 for upregulation and ≤ −0.58 for downregulation) and an adjusted *p*-value of ≤0.05. The protein and lipid had to be present in 5 of the 9 region-specific samples to be considered as a hit. The settings for the algorithm used in the joint-pathway analysis consisted of the following: we used “metabolic pathways” to determine pathways that contain both metabolites and the genes for the proteins. The Fisher’s Exact Test was used as enrichment analysis, topology was measured as degree centrality, and the p-values were based on a pathway level. All matched pathways were used for comparison among the altered region-specific pathways.

## Results

### Region-Specific Lipid and Protein Identification via MSI-Guided LC-MS/MS

The main workflow (MW) that we deployed combines the spatial information, provided by MALDI-MSI, with the structural information on LC-MS/MS. This approach enables the in-depth exploration of the lipid and protein profiles of distinct MSI-identified regions within one single tissue section. By using region-specific lipid and protein identifications instead of full section identifications, we enable additional insights into the biological relevance of specific regions within a tissue section. Two distinct segments were identified after performing segmentation on the MALDI-2-MSI data (Figure S1). H&E staining and pathological annotation of a consecutive GBM section confirmed the two segment identifications as tumor and necrotic regions. As such, two ROIs corresponding to tumor and necrosis were laser-dissected, extracted, and further analyzed after MSI analysis.

**Figure 2:**
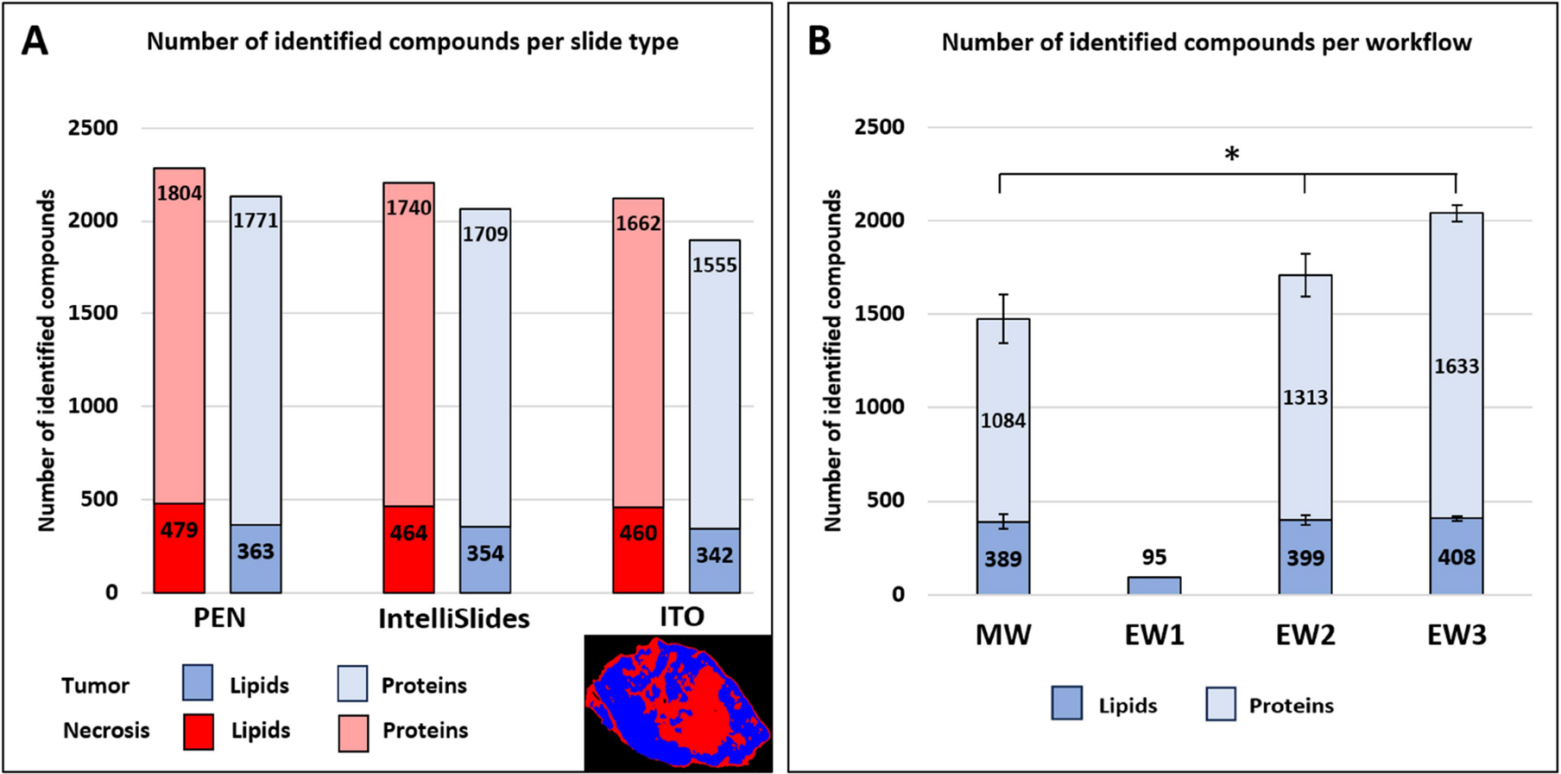
(A) Number of identified lipids and proteins for PEN-membrane-, IntelliSlides, and ITO-slides via single-section MSI-guided LC-MS/MS lipidomics and proteomics. The color of the bar corresponds to the colors used for the regions in the MSI segmentation image in the bottom right corner and Figure S1 (red = necrosis; blue = tumor). Lipids and proteins were considered to be identified only when detected in two samples of the indicated region. Lipid adducts and isotopes are removed. Data are presented as mean (*n* = 3). (B) Number of identified lipids and proteins per defined workflow. Results were based on PEN-membrane slides. Data are presented as mean ± SD (*n* = 3), * indicates significant differences to EW3 (*p*-value ≤ 0.05). Note that in EW2 and EW3, no spatial MS information is available.

The use of MSI-guided lipidomics and proteomics was assessed by the number of lipids and proteins identified by LC-MS/MS. PEN-membrane, IntelliSlides, and ITO-slides were compared for their potential use as slide types. 1 mm^2^ per ROI was extracted per slide, which resulted in approximately 350–480 lipid and 1550–1800 protein identifications depending on the slide type (Figure 2A). PEN-membrane slides provide overall the highest number of identifications. A more detailed overview of ROI-specific identification of the lipid classes is visualized in Table S2. Here, a low variation among the different lipid classes per slide type is observed, meaning that the choice of slide type does not significantly influence the identified lipid classes after LMD and LC-MS/MS analysis.

Next, we investigated the influence of the main workflow and the separately evaluated workflows on the number of identified lipids and proteins. Here, we compared the number of LC-MS/MS-identified lipids and proteins after MALDI-MSI (MW), after matrix deposition (EW2) and without any prior pretreatment (EW3). *m*/*z* values found via MALDI-MSI in EW1 are linked to *m*/*z* values, and lipid identifications are found via LC-MS/MS analysis. EW1 is exclusively based on the identification of lipids using MALDI-MSI. An overview of the number of identified lipids and proteins per workflow on PEN-membrane slides is shown in Figure 2B. Results show that matrix deposition and MALDI-MSI both significantly decrease the number of identified proteins compared to the workflow without any prior pretreatment.

### Comparison between Lipid Q-MSI and Q-LC-MS

**Figure 3:**
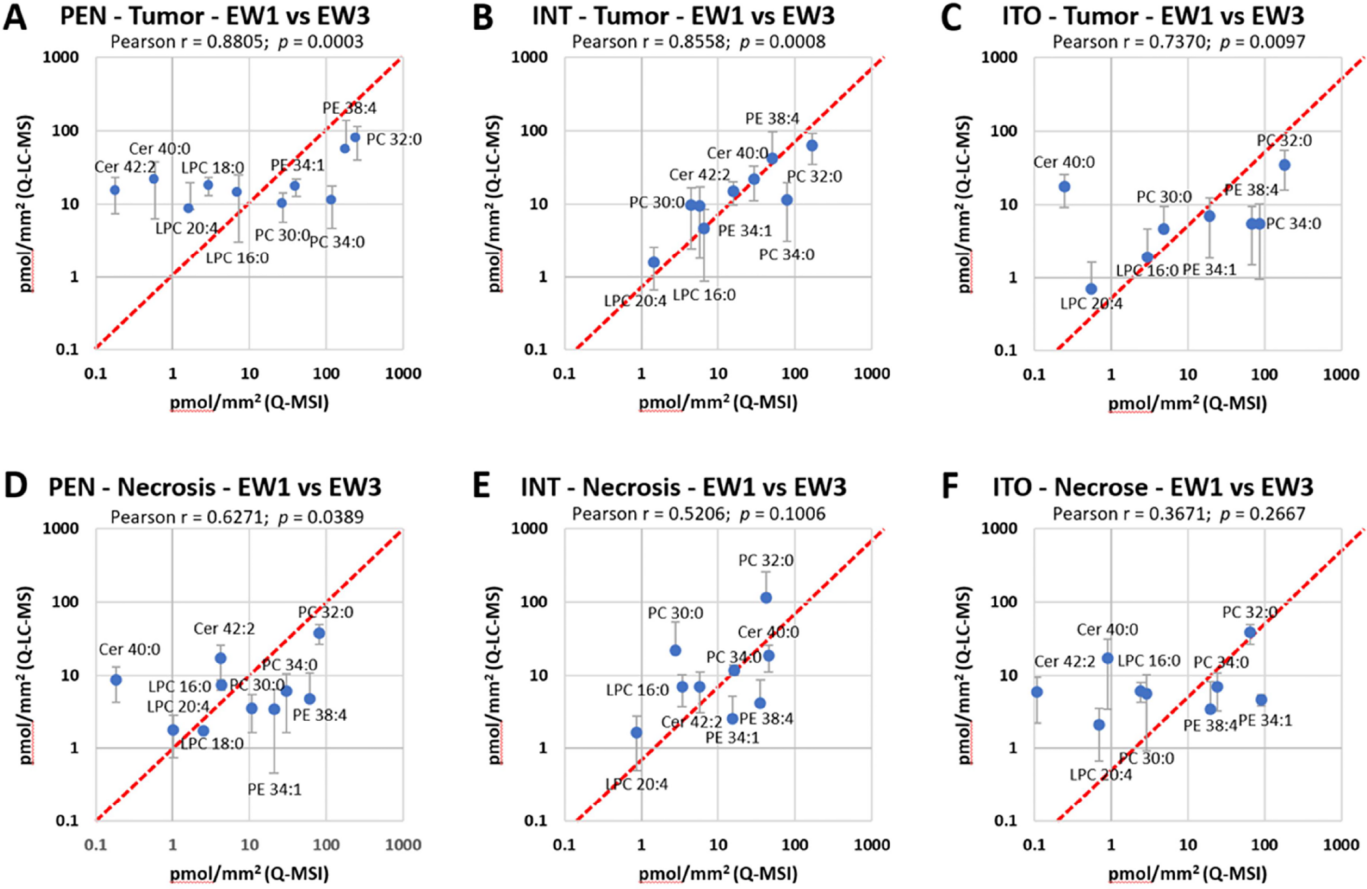
Pearson correlation of EW1 (direct Q-MSI) data and EW3 (direct Q-LC-MS) data of the tumor and necrotic region after summing the [M + H]^+^, [M + Na]^+^ and [M + K_39_]^+^ concentrations in pmol/mm^2^. (A,D) PEN-membrane, (B,E) IntelliSlides, and (C,F) ITO-slides. Each data point provides the corresponding lipid identifications. The red dotted line indicates a perfect correlation between Q-MSI and Q-LC-MS results.

Lipid quantification was assessed by comparing selected lipid concentrations in pmol/mm^2^. Comparison between Q-MSI and Q-LC-MS was carried out at three different levels:

First, the lipid quantification between two consecutive sections by using Q-MSI (EW1) and Q-LC-MS (EW3) was compared. In this experiment, the correlation between the quantitative values of the previously described protocol for Q-MSI, using an internal standard spray, was compared to Q-LC-MS.^7^ Q-LC-MS-based quantification is considered here as the golden standard. The Pearson correlation plots of Q-MSI and Q-LC-MS lipid concentrations in the tumor and necrotic region on the three different slide types (IntelliSlide, PEN-membrane, and ITO slides) are visualized in Figure 3. For all three slide types in both tumor and necrosis, we observe that Q-LC-MS results provide higher concentration values when measuring lipids in the low concentration range. This effect seems to diminish for lipids with higher concentrations, as concentration values for both Q-MSI and Q-LC-MS tend to meet, as seen by the data points converging to the red dotted line. Some data points deviate visibly more from the dotted line. These outliers, such as PC (32:0) and PC (34:0), were generally consistent among the different slide types. Here, the influence of the slide type can also be assessed by comparing the Pearson correlation values between the different slide types. The highest correlation is observed for PEN-membrane slides (Pearson *R* ≈ 0.62–0.88) compared to IntelliSlides (Pearson *R* ≈ 0.52–0.85) and ITO slides (Pearson *R* ≈ 0.36–0.73). The numeric Q-MSI and Q-LC-MS results are presented in Table S3.

Next, the possible effects of MSI measurements before Q-LC-MS were evaluated. Here, (Q-)MSI, LMD, extraction, and Q-LC-MS were performed on the same tissue section using PEN-membrane slides. The Pearson correlation plots of MSI-based quantification (EW1) and LC-MS-based quantification after MSI (MW) on the same tissue section are shown in Figure S2A,D. These results show that Q-MSI prior to Q-LC-MS analysis results in a correlation factor of Pearson *R* ≈ 0.85–0.93. Also, LC-MS-based quantification after MSI (MW) and LC-MS-based quantification without prior MSI (EW2) were compared to allow for the evaluation of the MSI effect itself on the lipid concentrations. Here, we are mostly interested in the overall performance of Q-LC-MS after performing MSI. These results were visualized in Figure S2B,E showing a Pearson correlation factor (Pearson *R* ≈ 0.94–0.95) when comparing MW and EW2.

Finally, to assess the effect of matrix and internal standard deposition on Q-LC-MS data, EW2 and EW3 were compared and visualized in Figure S2C,F. Pearson correlations of 0.90 and 0.91 were observed for necrosis and tumor, respectively. This indicates a strong positive correlation between the workflows in both necrosis and tumor, meaning that no significant effect of the matrix or internal standard deposition on lipid quantification is observed.

### Metabolite–Gene Interaction Network Analysis between Regions

The potential of the developed workflow for a single section was explored further by performing an interaction network analysis. This was accomplished on the significantly altered protein-encoding genes and lipids found in the different omics data from a single extraction. Table S4 shows the significantly altered metabolite–gene interaction pathways in the tumor region compared to the necrotic region. The distinctions between the up- and downregulated proteins and lipids are presented in Table S5. These findings align with previous observations of the tumor microenvironment. Glioblastoma cells, characterized by rapid proliferation, also require an upregulated glycerophospholipid metabolism.^16^ Additionally, these results identify an upregulated alanine, aspartate, and glutamate metabolism in the tumor microenvironment compared to the necrotic areas, highlighting the role of altered glutamine metabolism in gliomas.^17^ This analysis emphasizes the significance of integrating spatial information from MSI with the identification capabilities of LC-MS/MS to enhance a comprehensive understanding of metabolite–gene interaction networks across different regions.

## Discussion

We developed a main workflow to perform Q-MSI, qualitative and quantitative lipidomics, and proteomics in a single step extraction from molecularly and pathologically different regions on one single GBM section. The commonly used workflow up to this point, allowing for MSI-guided and LC-MS/MS-based spatial omics, is now extended with the ability to combine MSI-based lipid quantification with a single step lipid and protein extraction from the same dissected material. This is an important step for further insights into relevant biological processes. Indeed, single-section workflows are important to reduce the section-to-section variability and the sample volume that is required. In this workflow, we investigated the influence of the slide type, Q-MSI, and matrix deposition on the qualitative and quantitative lipidomics and proteomics results.

The qualitative LC-MS-based lipidomics and proteomics results show no significant difference when comparing slide types. Although it is expected that PEN-membrane slides give a higher number of lipid and protein identifications due to leaving a larger tissue area “intact” after ablation, this difference in identifications was not found in our study. A previously reported study, which compared the number of proteins after LMD on PEN-membrane, IntelliSlides, and ITO slides, showed a significant difference between the slide types. This study used a similar LMD-laser power, yet bigger aperture and slower laser speed. Both settings may cause more thermal tissue damage and influence the protein integrity, due to the longer exposure of the tissue to the laser.^11^ Based on other previously reported studies consistent with our work, it shows that an optimal laser setting for every sample type needs to be established.^2,18^

To identify the influence of MALDI-MSI and matrix deposition on the number of identified lipids and proteins, different workflows were evaluated. In Figure 2B, we observe a significantly higher number of proteins and lipids identified when no prior pretreatment was carried out on the section (EW3), meaning that MALDI-MSI (MW) negatively influenced both the number of lipid and protein identifications, whereas matrix deposition (EW2) negatively influenced the number of protein identifications. Since a lower number of protein identifications is observed in both workflows that contain matrix, it can be hypothesized that matrix suppresses the number of identifications in downstream proteomics. In order to limit matrix interferences in downstream processes such as LMD extraction or staining, the matrix is often washed away.^19^ In our study, the matrix that was deposited on the section could not be washed away, as this would result in the loss of the deposited lipid internal standard. Although the number of identified proteins in our main workflow is significantly lower compared to EW3, the number of identified proteins is still 10-fold higher compared to a previous study. This study uses the same extraction method, which reported approximately 100 identified proteins from 1 mm^2^ regions dissected by LMD on ITO and IntelliSlides.^11^ However, they used rat cardiac tissue for their extraction instead of human GBM, which is more biologically heterogeneous.

By assessing the comparison of Q-MSI and Q-LC-MS on three different levels, the difference between quantifications in Q-MSI and Q-LC-MS on the identical and consecutive sections was assessed. Q-LC-MS measured overall higher lipid concentrations when lipids were present in lower concentration ranges. This can be explained through the high ionization efficiency of electrospray ionization.^20^ Also, the chromatographic separation via LC can enhance the detection of low-concentration lipids by reducing sample complexity and minimizing interferences.^21^ Q-MSI, on the other hand, is valuable for its ability to provide spatial information about the distribution of lipids in a sample. However, the spatial information might come at the cost of sensitivity compared to Q-LC-MS, especially when dealing with low concentration lipids.^22^ As a consequence, it can be expected that lipids in higher concentration ranges have sufficient material for more accurate Q-MSI-based quantification compared to Q-LC-MS. This is due to the direct tissue analysis character of Q-MSI with a minimum compound loss in the matrix-based extraction step as compared to layer-based extraction in Q-LC-MS. Also, MALDI-2-MSI has a higher sensitivity for certain lipid species, such as PE, LPC, and ceramides, and can be affected by matrix effects.^23^ Here, the MALDI-2 laser and matrix, which are both used for ionization, can influence the ionization efficiency. This can impact the accuracy of the quantification. Since both methods use internal standards, the method of applying the internal standard mix is critical for obtaining reliable quantitative results.^24^ Via the use of internal standards, we expect to negate the ionization differences between MALDI and ESI.^25^ Internal standards help to correct for variations in ionization efficiency and matrix effects across different regions of the tissue, providing a more accurate quantification.^8,25,26^ The use of internal standards also enables the comparison of experiments between laboratories.^7^ Some of these benefits, however, still show difficulties and merit further investigation to fully understand the variances between MSI and LC-MS-based quantification using internal standards.

After reviewing the three different slide types, results showed that PEN-membrane slides give the highest correlation results when the Q-MSI and Q-LC-MS lipid concentrations. This can be related to the fact that the tissue stays more “intact” during ablation from PEN-membrane compared to other slide types, providing higher transferability of the tissue from the slide to the extraction container.^11^ The slides also have different surface coatings that can affect the adhesion of the tissue samples during microdissection. Since we see different correlation factors, it can be hypothesized that the type of coating can impact the extraction efficiency of lipids and subsequently affect the quantification correlation factors. It was described before that PEN-membrane slides have specific features that make them more suitable for LMD compared to others.^18^

Since MSI prior to Q-LC-MS is currently considered as the standard operating protocol for lipid quantification in mass spectrometry imaging workflows, we evaluated the effect of MSI on LC-MS-based lipid quantifications. These experiments were all performed on PEN-membrane slides, as these gave the highest correlation factor in comparison to the different slide types. When comparing the effect of MSI on Q-LC-MS, we observed that performing MSI and Q-LC-MS on the same section results in a low Pearson correlation, which can be explained due to the semidestructiveness of the MALDI-laser. Since the internal standard is sprayed between the sample and matrix, it can be expected that when the MALDI-laser hits the sample, part of the sample and internal standard mixture is already ablated and thus eradicated for downstream analysis. We also observed that spraying the internal standard and matrix on the tissue barely affects the lipid quantification results in Q-LC-MS. Since high Pearson correlations (Pearson *R*: 0.90–0.91) were observed, it can be stated that this does not influence the lipid quantification. This method of homogeneously spraying the internal standard can be introduced as a new way of applying internal standard on a tissue section, compared to other methods such as internal standard spotting.^27^

By performing a region-specific metabolite–gene network analysis, we showed the versatility of the proposed workflow. The significance of region-specific metabolite–gene pathway analysis compared to bulk approaches lies in its ability to unveil the complicated and specialized workings of cells and tissues. While bulk analysis provides a global overview of cellular processes, it often overlooks the diversity within distinct regions of a tissue. By obtaining the altered proteins and lipids and performing a metabolite–gene analysis, we were able to visualize the different and overlapping pathways that were significantly altered in the tumor region compared to the necrotic region. To briefly discuss a few, altered glycerolipid and glycerophospholipid metabolism is a common feature of cancer cells, including glioblastoma. Changes in the synthesis and breakdown of these lipids can impact the composition cell membranes, signaling pathways, and energy storage.^28^ Our results also show alterations in the arginine biosynthesis, nicotinate and nicotinamide metabolism, pyruvate metabolism, and sphingolipid metabolism. These pathways have all been previously described as important pathways in glioblastoma.^29^ Interestingly, we see that the porphyrin and chlorophyll metabolism pathway in tumor is downregulated when compared to necrosis. 5-aminolevulinic acid (5-ALA), an amino acid administered before fluorescence-guided resection of glioblastomas, is a porphyrin that is metabolized by cells where the heme-synthesis is activated.^30^ Heme-synthesis induces programmed necrosis in macrophages, which are known to accumulate 5-ALA.^31^ There is also evidence that 5-ALA destroys vascular endothelial cells, which indirectly contributes to necrosis.^32^ These pathways show that region-localized lipid–protein interactions are important to understand the extent of heterogeneity in a tissue. This again indicates that MALDI-MSI-based region extraction combined with complementary proteomic and lipidomic information is of great added value in understanding diseases.

## Conclusions

This study resulted in a dedicated workflow that enables a combined MSI-LC-MS/MS single step extraction analysis for proteomics and quantitative lipidomics on a single tissue section. This workflow starts by spraying a tissue section on a PEN-membrane slide with internal standard and matrix and consecutively performing Q-MSI, MSI-guided laser-capture microdissection and a single step lipidomic and proteomic extraction. Q-LC-MS(/MS) is used for quantification and identification. This main workflow, compared with our other evaluated workflows, encompasses the needed output for a comprehensive molecular overview of a single section. Indeed, it allows for lipid localization and quantification as well as identification of lipids and proteins in a selected area of a single tissue section. Our findings showed the critical importance of cautious interpretation when dealing with Q-MSI data. It is also important to note that MSI prior to Q-LC-MS on the same tissue section results in a lower number of protein identifications. Therefore, depending on the research question, we recommend acquiring the full proteomic profile on a consecutive section whenever enough material is available.

In our single-section workflow, we demonstrate the ability to perform a single extraction after image-guided dissection that can be used for both lipidomic and proteomic analysis. Therefore, it is possible to look at significantly altered proteins and lipids between regions within a single section, in our case, GBM, eliminating the need for multiple sections when conducting combined lipidomic and proteomic studies. As a result, lipid–protein interaction networks allow for a great expansion of biological information from a single tissue section.

In conclusion, we showed the effects of the different steps in a single workflow on the lipid identification and quantification, as well as the protein identification. Based on these findings, we propose a workflow for the comprehensive elucidation of molecular information from a single section. We address several important caveats that need to be taken into consideration. Our work will lead to a significant increase in multiomics information from a single tissue section. Moreover, better and more carefully stated conclusions from single-section lipidomics and proteomics can be taken, leading to better insights into disease-related pathways.

## Supporting Information Available

Internal standard and matrix spraying parameters; hematoxylin and eosin staining; settings laser capture microdissection; H&E, MSI and segmentation image; Pearson correlation plots of evaluated workflows; internal standard composition; region-specific lipids per class; lipid quantifications Q-MSI and Q-LC-MS/MS; altered metabolite–gene pathways; and altered proteins and lipids (PDF)

## References

[ref1] Irie M.; Fujimura Y.; Yamato M.; Miura D.; Wariishi H. Integrated MALDI-MS imaging and LC-MS techniques for visualizing spatiotemporal metabolomic dynamics in a rat stroke model. Metabolomics 2014, 10 (3), 473–483. 10.1007/s11306-013-0588-8. 24772057 PMC3984668

[ref2] Dewez F.; Martin-Lorenzo M.; Herfs M.; Baiwir D.; Mazzucchelli G.; De Pauw E.; Heeren R. M. A.; Balluff B. Precise co-registration of mass spectrometry imaging, histology, and laser microdissection-based omics. Anal Bioanal Chem. 2019, 411 (22), 5647–5653. 10.1007/s00216-019-01983-z. 31263919 PMC6704276

[ref3] aGilard V.; Ferey J.; Marguet F.; Fontanilles M.; Ducatez F.; Pilon C.; Lesueur C.; Pereira T.; Basset C.; Schmitz-Afonso I.; et al. Integrative Metabolomics Reveals Deep Tissue and Systemic Metabolic Remodeling in Glioblastoma. Cancers (Basel) 2021, 13 (20), 5157 10.3390/cancers13205157. 34680306 PMC8534284

[ref4] Eiersbrock F. B.; Orthen J. M.; Soltwisch J. Validation of MALDI-MS imaging data of selected membrane lipids in murine brain with and without laser postionization by quantitative nano-HPLC-MS using laser microdissection. Anal Bioanal Chem. 2020, 412 (25), 6875–6886. 10.1007/s00216-020-02818-y. 32712813 PMC7496020

[ref5] Unsihuay D.; Mesa Sanchez D.; Laskin J. Quantitative Mass Spectrometry Imaging of Biological Systems. Annu. Rev. Phys. Chem. 2021, 72, 307–329. 10.1146/annurev-physchem-061020-053416. 33441032 PMC8161172

[ref6] Dewez F.; Oejten J.; Henkel C.; Hebeler R.; Neuweger H.; De Pauw E.; Heeren R. M. A.; Balluff B. MS Imaging-Guided Microproteomics for Spatial Omics on a Single Instrument. Proteomics 2020, 20 (23), e1900369 10.1002/pmic.201900369. 32767647

[ref7] Vandenbosch M.; Mutuku S. M.; Mantas M. J. Q.; Patterson N. H.; Hallmark T.; Claesen M.; Heeren R. M. A.; Hatcher N. G.; Verbeeck N.; Ekroos K.; et al. Toward Omics-Scale Quantitative Mass Spectrometry Imaging of Lipids in Brain Tissue Using a Multiclass Internal Standard Mixture. Anal. Chem. 2023, 95, 18719 10.1021/acs.analchem.3c02724. 38079536 PMC11372745

[ref8] aTaylor A. J.; Dexter A.; Bunch J. Exploring Ion Suppression in Mass Spectrometry Imaging of a Heterogeneous Tissue. Anal. Chem. 2018, 90 (9), 5637–5645. 10.1021/acs.analchem.7b05005. 29461803

[ref9] Louis D. N.; Perry A.; Wesseling P.; Brat D. J.; Cree I. A.; Figarella-Branger D.; Hawkins C.; Ng H. K.; Pfister S. M.; Reifenberger G.; et al. The 2021 WHO Classification of Tumors of the Central Nervous System: a summary. Neuro Oncol 2021, 23 (8), 1231–1251. 10.1093/neuonc/noab106. 34185076 PMC8328013

[ref10] O’Neill K. C.; Liapis E.; Harris B. T.; Perlin D. S.; Carter C. L. Mass spectrometry imaging discriminates glioblastoma tumor cell subpopulations and different microvascular formations based on their lipid profiles. Sci. Rep 2022, 12 (1), 17069 10.1038/s41598-022-22093-4. 36224354 PMC9556690

[ref11] Mezger S. T. P.; Mingels A. M. A.; Bekers O.; Heeren R. M. A.; Cillero-Pastor B. Mass Spectrometry Spatial-Omics on a Single Conductive Slide. Anal. Chem. 2021, 93 (4), 2527–2533. 10.1021/acs.analchem.0c04572. 33412004 PMC7859928

[ref12] Liu H.; Qiu W.; Sun T.; Wang L.; Du C.; Hu Y.; Liu W.; Feng F.; Chen Y.; Sun H. Therapeutic strategies of glioblastoma (GBM): The current advances in the molecular targets and bioactive small molecule compounds. Acta Pharm. Sin B 2022, 12 (4), 1781–1804. 10.1016/j.apsb.2021.12.019. 35847506 PMC9279645

[ref13] Soltwisch J.; Kettling H.; Vens-Cappell S.; Wiegelmann M.; Muthing J.; Dreisewerd K. Mass spectrometry imaging with laser-induced postionization. Science 2015, 348 (6231), 211–215. 10.1126/science.aaa1051. 25745064

[ref14] Breitkopf S. B.; Ricoult S. J. H.; Yuan M.; Xu Y.; Peake D. A.; Manning B. D.; Asara J. M. A relative quantitative positive/negative ion switching method for untargeted lipidomics via high resolution LC-MS/MS from any biological source. Metabolomics 2017, 13 (3), 1 10.1007/s11306-016-1157-8. 28496395 PMC5421409

[ref15] Tortorella S.; Tiberi P.; Bowman A. P.; Claes B. S. R.; Scupakova K.; Heeren R. M. A.; Ellis S. R.; Cruciani G. LipostarMSI: Comprehensive, Vendor-Neutral Software for Visualization, Data Analysis, and Automated Molecular Identification in Mass Spectrometry Imaging. J. Am. Soc. Mass Spectrom. 2020, 31 (1), 155–163. 10.1021/jasms.9b00034. 32881505

[ref16] avan Meer G.; Voelker D. R.; Feigenson G. W. Membrane lipids: where they are and how they behave. Nat. Rev. Mol. Cell Biol. 2008, 9 (2), 112–124. 10.1038/nrm2330. 18216768 PMC2642958

[ref17] aFirdous S.; Abid R.; Nawaz Z.; Bukhari F.; Anwer A.; Cheng L. L.; Sadaf S. Dysregulated Alanine as a Potential Predictive Marker of Glioma-An Insight from Untargeted HRMAS-NMR and Machine Learning Data. Metabolites 2021, 11 (8), 507 10.3390/metabo11080507. 34436448 PMC8402070

[ref18] Dilillo M.; Pellegrini D.; Ait-Belkacem R.; de Graaf E. L.; Caleo M.; McDonnell L. A. Mass Spectrometry Imaging, Laser Capture Microdissection, and LC-MS/MS of the Same Tissue Section. J. Proteome Res. 2017, 16 (8), 2993–3001. 10.1021/acs.jproteome.7b00284. 28648079

[ref19] Zaima N.; Hayasaka T.; Goto-Inoue N.; Setou M. Matrix-assisted laser desorption/ionization imaging mass spectrometry. Int. J. Mol. Sci. 2010, 11 (12), 5040–5055. 10.3390/ijms11125040. 21614190 PMC3100838

[ref20] Koivusalo M.; Haimi P.; Heikinheimo L.; Kostiainen R.; Somerharju P. Quantitative determination of phospholipid compositions by ESI-MS: effects of acyl chain length, unsaturation, and lipid concentration on instrument response. J. Lipid Res. 2001, 42 (4), 663–672. 10.1016/S0022-2275(20)31176-7. 11290839

[ref21] Fauland A.; Kofeler H.; Trotzmuller M.; Knopf A.; Hartler J.; Eberl A.; Chitraju C.; Lankmayr E.; Spener F. A comprehensive method for lipid profiling by liquid chromatography-ion cyclotron resonance mass spectrometry. J. Lipid Res. 2011, 52 (12), 2314–2322. 10.1194/jlr.D016550. 21960706 PMC3220297

[ref22] Li D.; Ouyang Z.; Ma X. Mass Spectrometry Imaging for Single-Cell or Subcellular Lipidomics: A Review of Recent Advancements and Future Development. Molecules 2023, 28 (6), 2712 10.3390/molecules28062712. 36985684 PMC10057629

[ref23] Bowman A. P.; Bogie J. F. J.; Hendriks J. J. A.; Haidar M.; Belov M.; Heeren R. M. A.; Ellis S. R. Evaluation of lipid coverage and high spatial resolution MALDI-imaging capabilities of oversampling combined with laser post-ionisation. Anal Bioanal Chem. 2020, 412 (10), 2277–2289. 10.1007/s00216-019-02290-3. 31879798 PMC7118047

[ref24] Holbrook J. H.; Kemper G. E.; Hummon A. B. Quantitative mass spectrometry imaging: therapeutics & biomolecules. Chem. Commun. (Camb) 2024, 60, 2137 10.1039/D3CC05988J. 38284765 PMC10878071

[ref25] Dewez F.; De Pauw E.; Heeren R. M. A.; Balluff B. Multilabel Per-Pixel Quantitation in Mass Spectrometry Imaging. Anal. Chem. 2021, 93 (3), 1393–1400. 10.1021/acs.analchem.0c03186. 33373197 PMC7871324

[ref26] Tobias F.; Hummon A. B. Considerations for MALDI-Based Quantitative Mass Spectrometry Imaging Studies. J. Proteome Res. 2020, 19 (9), 3620–3630. 10.1021/acs.jproteome.0c00443. 32786684 PMC8221076

[ref27] Perez C. J.; Ifa D. R. Internal standard application strategies in mass spectrometry imaging by desorption electrospray ionization mass spectrometry. Rapid Commun. Mass Spectrom. 2021, 35 (8), e9053 10.1002/rcm.9053. 33474774

[ref28] Wang Z.; Zhang Z.; Zhang K.; Zhou Q.; Chen S.; Zheng H.; Wang G.; Cai S.; Wang F.; Li S. Multi-Omics Characterization of a Glycerolipid Metabolism-Related Gene Enrichment Score in Colon Cancer. Front Oncol 2022, 12, 881953 10.3389/fonc.2022.881953. 35600382 PMC9117699

[ref29] aHou X.; Chen S.; Zhang P.; Guo D.; Wang B. Targeted Arginine Metabolism Therapy: A Dilemma in Glioma Treatment. Front Oncol 2022, 12, 938847 10.3389/fonc.2022.938847. 35898872 PMC9313538

[ref30] Smith S. J.; Rowlinson J.; Estevez-Cebrero M.; Onion D.; Ritchie A.; Clarke P.; Wood K.; Diksin M.; Lourdusamy A.; Grundy R. G.; et al. Metabolism-based isolation of invasive glioblastoma cells with specific gene signatures and tumorigenic potential. Neurooncol Adv. 2020, 2 (1), vdaa087 10.1093/noajnl/vdaa087. 32904996 PMC7462276

[ref31] Traylor J. I.; Pernik M. N.; Sternisha A. C.; McBrayer S. K.; Abdullah K. G. Molecular and Metabolic Mechanisms Underlying Selective 5-Aminolevulinic Acid-Induced Fluorescence in Gliomas. Cancers 2021, 13 (3), 580 10.3390/cancers13030580. 33540759 PMC7867275

[ref32] Chang C. J.; Sun C. H.; Liaw L. H.; Berns M. W.; Nelson J. S. In vitro and in vivo photosensitizing capabilities of 5-ALA versus photofrin in vascular endothelial cells. Lasers Surg Med. 1999, 24 (3), 178–186. 10.1002/(SICI)1096-9101(1999)24:3<178::AID-LSM2>3.0.CO;2-W. 10229148

