# Supplementary material for: MALDI-MSI-LC-MS/MS Workflow for Single-Section Single Step Combined Proteomics and Quantitative Lipidomics
Source: Anal Chem. 2024 Mar 4;96(10):4266–74. doi: 10.1021/acs.analchem.3c05850 (PMC10938281; doi:10.1021/acs.analchem.3c05850)
Supplement: Supplementary file 1 — ac3c05850_si_001.pdf [file ac3c05850_si_001.pdf]

Supporting information:

MALDI-MSI-LC-MS/MS Workflow for Single Section - Single Step Combined Proteomics and Quantitative Lipidomics

Tim F.E. Hendriks[1], Kasper K. Krestensen[1], Ronny Mohren[1], Michiel Vandenbosch[1], Steven De Vleeschouwer[2], Ron M.A. Heeren[1], Eva Cuypers[1]*

[1]The Maastricht MultiModal Molecular Imaging (M4I) institute, Division of Imaging Mass Spectrometry (IMS), Maastricht University, 6229 ER Maastricht, The Netherlands

[2]Department of Neurosurgery, Laboratory for Experimental Neurosurgery and Neuroanatomy, UZ Leuven, KU Leuven, 3000 Leuven, Belgium

*Corresponding author: e.cuypers@maastrichtuniversity.nl

Supporting Information Content

Spraying parameters........3
Hematoxylin and eosin staining........3
Laser Capture Microdissection (LMD)........3
Supplementary figure 1: H&E, MSI and segmentation image........4
Supplementary figure 2: Pearson correlation plots of evaluated workflows........5
Supplementary table 1: Internal standard composition........6
Supplementary table 2: Region-specific lipids per class........7
Supplementary table 3: Lipid quantifications Q-MSI and Q-LC-MS/MS........8
Supplementary table 4: Altered metabolite-gene pathways........9
Supplementary table 5: Altered proteins and lipids........10

Spraying parameters

The internal standard dilution was sprayed with an off-line 2.5 mL syringe (Trajan Scientific, Victoria, Australia) in combination with a syringe pump connected to a HTX TM-Sprayer (HTX Technologies LLC). Spraying parameters for the internal standard were as followed: temperature = 30°C, flow rate = 0.06 mL/min, velocity = 1200 mm/min, track spacing = 3 mm, gas flow = 2 L/min, N2 gas pressure = 10 psi nozzle height = 40 mm, drying time = 30 s, number of passes = 16, in a C-C pattern. The concentration of the internal standard in picomole per squared millimeter (pmol/mm2) was calculated by multiplying the concentration of the internal standard (µg/mL), flow rate (mL/min), time (min), number of passes (layers) divided by the surface area sprayed (mm2). This number was then divided by dilution factor and the molecular weight (Da), resulting in a value in pmol/mm2. After applying the internal standard, the samples were sprayed with 15 mg/mL 2,5-DHB diluted in 2:1 CHCl3:MeOH. Spraying parameters for 2,5-DHB were as followed: temperature = 50°C, flow rate = 0.12 mL/min, velocity = 1200 mm/min, track spacing = 3 mm, gas flow = 2 L/min, N2 gas pressure = 10 psi, nozzle height = 40 mm, drying time = 30 s, number of passes = 10, in a C-C pattern.

Hematoxylin and eosin staining

Hematoxylin and eosin (H&E) staining was performed on consecutive sections to the sections used in the MALDI-MSI experiments. The H&E staining procedure was performed as followed: the slides were hydrated in water for 1 minute, stained in hematoxylin for 3 minutes, rinsed with running tap water for 3 minutes, stained in eosin staining for 30 seconds, and again rinsed with running tap water for 3 minutes. The slides were then immersed in 100% ethanol for 1 minute, afterwards xylene washed for 2 minutes, and Entellan-mounted carefully with a coverslip, and left to air-dry at room temperature. All optical images were captured at high resolution using the Leica AperioCS2 scanner (20x objective) via Aperio ImageScope software (version 12.4.3.5008) from Leica Biosystems Imaging (Germany).

Laser Capture Microdissection (LMD)

The coordinate information of the ROIs were exported via LipostarMSI to a Leica LMD 7000 (Leica Microsystems) for laser capture microdissection. The ROIs were dissected using the following laser parameters: power = 54, aperture = 10, speed = 30, specimen balance = 25, line spacing = 5, head current at 100% and pulse frequency at 250 Hz in draw + scan mode for ITO-slides and Intellislides. Laser parameters for PEN-membrane slides were power = 40, aperture = 7, speed = 10, specimen balance = 0, line spacing = 29, head current at 100% and pulse frequency at 2600 Hz in draw + cut mode. Directly after dissection the tissue was collected in the cap of 0.5 mL sterilized PCR-tubes, pre-filled with 20 µL MeOH. The dissected material was transferred to a 2 mL Eppendorf tube and the cap was washed four times with 20 µL MeOH, for a total of 100 µL MeOH.

Supplementary figure 1

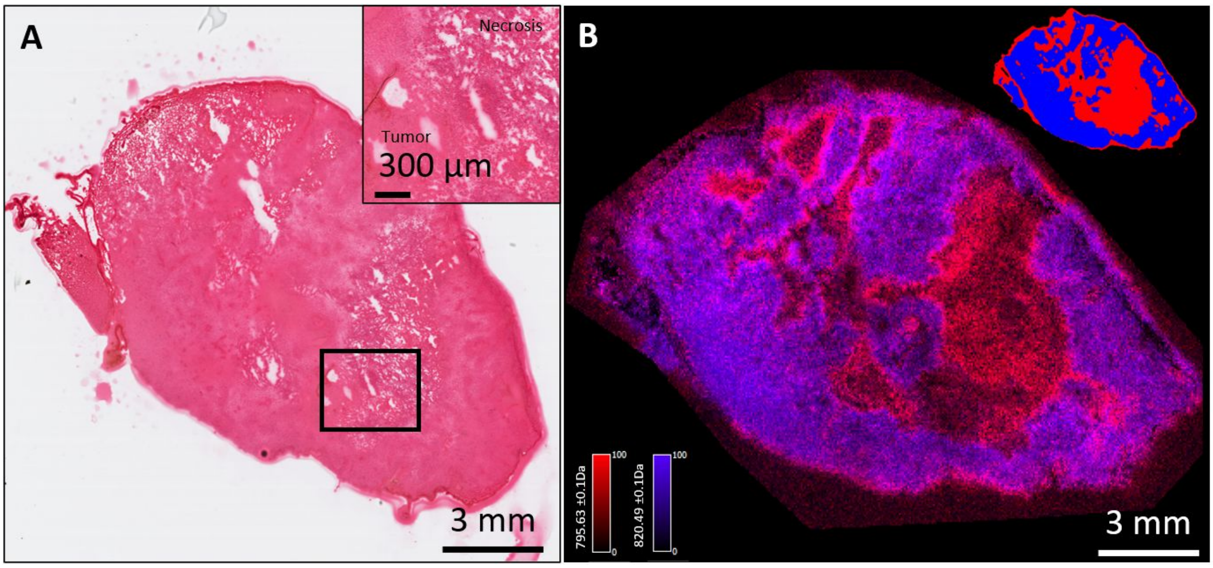

Supplementary figure 1. (A) H&E stained GBM section with zoomed in annotated region. An experienced pathologist defined the tumor and necrotic regions. (B) MALDI-2-MSI image of m/z 795.63 and 820.49 corresponding to the necrotic and tumor region respectively. Top corner shows the MSI-segmentation image with necrosis in red and tumor in blue, based on all peaks between m/z 600 – 1000.

Supplementary figure 2

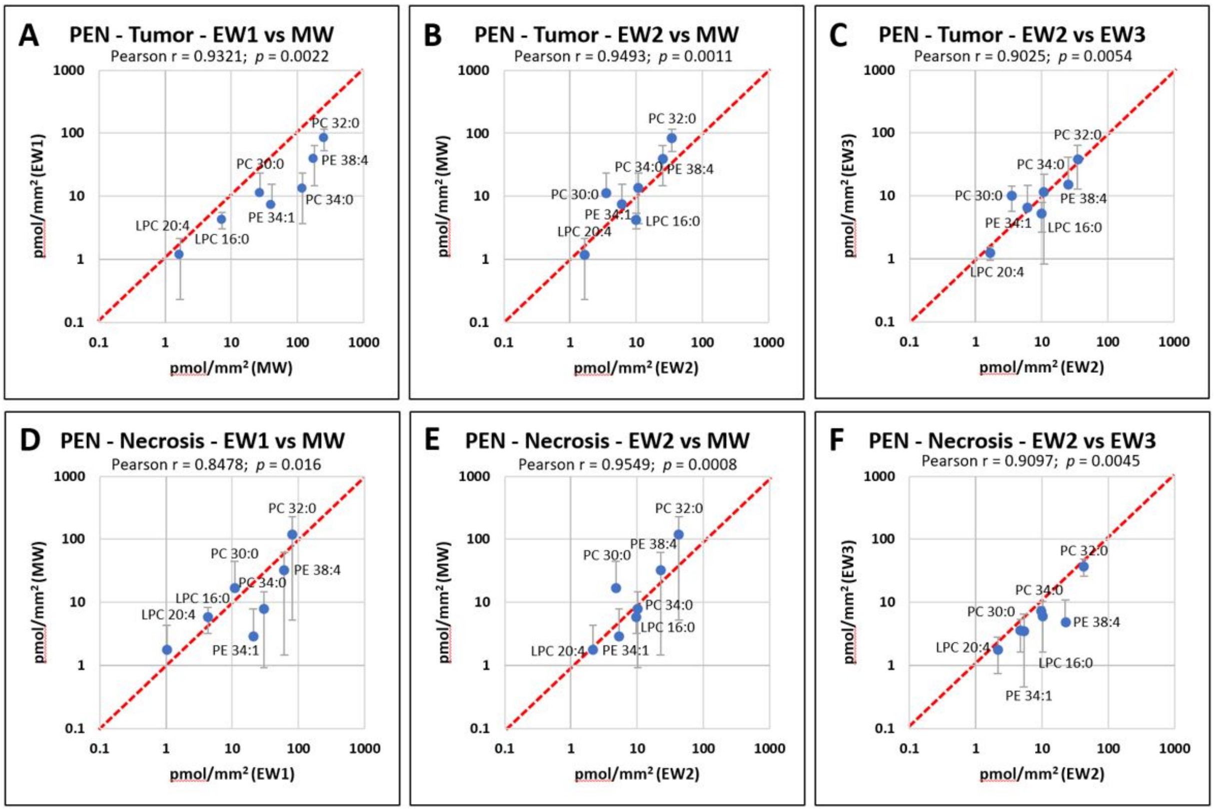

Supplementary figure 2. Pearson correlation of Q-MSI and Q-LC-MS data after accumulating the $[M+H]^+$, $[M+Na]^+$ and $[M+K_{39}]^+$ concentrations in pmol/mm^2 for PEN-membrane for tumor and necrosis respectively. In (A&D) EW1 (direct Q-MSI) and the main workflow (Q-LC-MS after imaging) are compared. In (B&E) the quantifications of EW2 (Q-LC-MS without imaging) and the main workflow (Q-LC-MS after imaging) are compared. (C&F) shows the comparison of the lipid quantification between EW2 and EW3 (direct Q-LC-MS). The red dotted line indicates a perfect correlation between Q-MSI and Q-LC-MS results.

Supplementary table 1

MSI SPLASH mix internal standard composition and concentrations for MALDI-2-MSI positive ionization mode. Internal standards in **bold** were detected via MALDI-2-MSI.

Name	Molecular Formula	Average Mass (Da)	[ISTD]	[Sprayed]	[Tissue]
			(µg/mL)	(µg/mm2)	pmol/mm2
**15:0-18:1 (d7) PC**	**$C_{41}H_{73}D_7NO_8P$**	**753.093**	**161**	**0.208**	**27.66**
**15:0-18:1 (d7) PE**	**$C_{38}H_{67}D_7NO_8P$**	**711.012**	**100**	**0.129**	**18.2**
**17:0 (d5) Lyso PC**	**$C_{25}H_{47}D_5NO_7P$**	**514.687**	**3**	**0.004**	**0.75**
17:0 (d5) Lyso PE	$C_{22}H_{41}D_5NO_7P$	472.607	3	0.004	0.82
**18:1-18:1 SM (d9)**	**$C_{41}H_{72}D_9N_2O_6P$**	**738.12**	**31**	**0.04**	**5.43**
C15 Lactosyl(β) Ceramide (d18:1-d7/15:0)	$C_{45}H_{78}D_7NO_{13}$	855.198	13	0.017	1.97
**C18 Ceramide-d7 (d18:1/18:0)**	**$C_{36}H_{64}D_7NO_3$**	**572.996**	**11**	**0.014**	**2.48**
**C17 Glucosyl(β) Ceramide (d18:1/17:0)**	**$C_{41}H_{79}NO_8$**	**714.068**	**133**	**0.172**	**24.1**

Supplementary table 2

Number of identified region-specific lipids per lipid class on different slide types. Lipid adducts and isotopes are removed from the data set. Lipids had to be found in at least two samples related to the ROI and slide to be included for lipid identification.

	PEN		IntelliSlides		ITO	
**Lipid Class**	**Necrosis**	**Tumor**	**Necrosis**	**Tumor**	**Necrosis**	**Tumor**
1-(1Z-alkenyl),2-acylglycerophosphocholines	5	7	5	7	5	7
1-(1Z-alkenyl),2-acylglycerophosphoethanolamines	4	7	4	7	4	6
1-alkyl,2-acylglycerophosphocholines	22	19	19	22	19	19
1-alkyl,2-acylglycerophosphoethanolamines	2	2	2	2	2	2
C21 steroids (gluco/mineralocorticoids, progestogins) and derivatives	6	8	7	8	7	7
Ceramide phosphocholines (sphingomyelins)	33	28	33	28	33	28
Cholesterol and derivatives	1	1	1	1	1	1
Diacylglycerols	30	33	31	32	28	32
Diacylglycerophosphates	2	1	2	1	2	1
Diacylglycerophosphocholines	53	46	46	44	49	42
Diacylglycerophosphoethanolamines	11	11	8	10	7	7
Diacylglycerophosphoglycerols	1	1	1	1	1	1
Diacylglycerophosphoinositols	1	2	1	2	0	0
Diacylglycerophosphoserines	1	2	1	1	1	1
Ergosterols and C24-methyl derivatives	1	1	1	1	1	1
Fatty acyl carnitines	5	5	4	5	4	4
Gal- (Gala series)	2	0	2	0	2	0
Glycine conjugates	1	1	1	1	1	1
Monoacylglycerophosphocholines	5	5	5	5	4	5
Monoacylglycerophosphoethanolamines	5	5	5	5	4	5
N-acyl ethanolamines (endocannabinoids)	1	1	1	1	1	1
N-acyl-4-hydroxysphinganines (phytoceramides)	7	7	6	7	7	7
N-acylsphinganines (dihydroceramides)	18	16	18	16	17	16
N-acylsphingosines (ceramides)	6	7	6	6	5	6
Prostaglandins	2	2	2	2	2	2
Simple Glc series	1	1	1	1	1	1
Sphinganines	1	1	1	1	1	1
Sphingoid base analogs	3	3	3	3	3	3
Sphingoid base homologs and variants	3	3	3	3	3	3
Steryl esters	7	7	6	6	6	4
Stigmasterols and C24-ethyl derivatives	3	3	2	2	3	3
Triacylglycerols	236	127	236	123	236	125

Supplementary table 3

List of lipids and their quantification in pmol/mm² for comparing Q-MSI and Q-LC-MS between different slide types and tissue regions. Concentrations are a sum of the $[M+H]^+$, $[M+Na]^+$ and $[M+K_{39}]^+$ adducts. The corresponding internal standard is shown for each lipid class. **N.F.** = Lipid not found in Lipostar LC-MS identification database.

					**ISTD sprayed**	**MSI PEN**	**LC-MS PEN**	**MSI Int**	**LC-MS Int**	**MSI ITO**	**LC-MS ITO**
	**Lipid ID**	**m/z**	**ISTD ID**	**m/z**	µg/mm²	pmol/mm²	pmol/mm²	pmol/mm²	pmol/mm²	pmol/mm²	pmol/mm²
**Tumor**	**PC 30:0**	705.54				27.2	9.9	4.5	9.6	4.9	4.6
	**PC 32:0**	733.57	**PC 33:1d7**	752.61	30.5	251.5	77.5	168.4	63.9	181.7	35.0
	**PC 34:0**	761.59				118.3	11.1	78.1	11.5	85.6	5.5
	**PE 34:0**	719.55				99.6	**N.F.**	68.8	**N.F.**	87.2	**N.F.**
	**PE 34:1**	739.52	**PE 33:1d7**	741.42	20.1	40.6	17.4	15.6	14.8	19.3	7.0
	**PE 38:4**	767.55				180.3	55.4	51.0	42.4	68.1	5.4
	**LPC 16:0**	495.34				7.2	13.9	6.6	4.6	3.0	1.9
	**LPC 18:0**	523.37	**LPC 17:0d5**	547.31	0.9	2.9	18.0	1.9	2.5	1.9	6.0
	**LPC 20:4**	544.34				1.7	8.5	1.4	1.5	0.6	0.7
	**Cer (d40:0)**	630.61				4.6	15.2	10.6	1.9	12.1	6.3
	**Cer (42:2)**	640.60	**Cer (d36:1)**	573.59	2.9	0.2	21.9	5.8	9.4	5.17	8.3
	**Cer (40:0)**	652.59				0.5	**N.F.**	29.1	22.1	30.9	17.4
	**HexCer36:2**	726.55				49.05	**N.F.**	815.7	**N.F.**	652.9	**N.F.**
	**HexCer36:1**	728.56	**HexCer (d35:1)**	714.58	27.64	8.79	**N.F.**	85.0	**N.F.**	66.3	**N.F.**
	**HexCer42:2**	810.65				5.8	**N.F.**	84.5	**N.F.**	65.9	**N.F.**
	**SM 34:1**	703.57				60.1	**N.F.**	37.5	**N.F.**	207.6	**N.F.**
	**SM 36:2**	729.59	**SM (d36:2)**	738.64	6.3	24.2	**N.F.**	17.9	**N.F.**	101.3	**N.F.**
	**SM 36:1**	730.59				11.2	**N.F.**	8.4	**N.F.**	50.48	**N.F.**
**Necrosis**	**PC 30:0**	705.54				10.8	3.5	2.8	21.6	2.9	5.6
	**PC 32:0**	733.57	**PC 33:1d7**	752.61	30.5	80.4	37.4	41.9	115.5	64.5	38.2
	**PC 34:0**	761.59				30.2	6.0	16.0	11.6	24.2	7.0
	**PE 34:0**	719.55				109.9	**N.F.**	66.4	**N.F.**	91.1	**N.F.**
	**PE 34:1**	739.52	**PE 33:1d7**	741.42	20.1	20.8	3.4	15.4	2.5	19.8	4.6
	**PE 38:4**	767.55				61.1	4.7	35.4	4.1	63.6	3.3
	**LPC 16:0**	495.34				4.3	7.3	3.4	6.9	2.4	6.1
	**LPC 18:0**	523.37	**LPC 17:0d5**	547.31	0.9	2.5	1.7	1.6	2.8	2.0	6.0
	**LPC 20:4**	544.34				1.0	1.7	0.9	1.6	0.7	2.1
	**Cer (d40:0)**	630.61				4.2	7.5	14.6	3.7	23.9	5.8
	**Cer (42:2)**	640.60	**Cer (d36:1)**	573.59	2.9	0.2	4.2	5.8	7.0	8.9	17.0
	**Cer (40:0)**	652.59				0.2	8.5	45.1	18.4	75.0	**N.F.**
	**HexCer36:2**	726.55				101.4	**N.F.**	2020.2	**N.F.**	2338	**N.F.**
	**HexCer36:1**	728.56	**HexCer (d35:1)**	714.58	27.64	16.1	**N.F.**	226.6	**N.F.**	266.3	**N.F.**
	**HexCer42:2**	810.65				11.1	**N.F.**	213.3	**N.F.**	247.5	**N.F.**
	**SM 34:1**	703.57				69.7	**N.F.**	25.6	**N.F.**	241.7	**N.F.**
	**SM 36:2**	729.59	**SM (d36:2)**	738.64	6.3	13.5	**N.F.**	6.4	**N.F.**	61.8	**N.F.**
	**SM 36:1**	730.59				6.4	**N.F.**	3.1	**N.F.**	30.4	**N.F.**

Supplementary table 4

Significantly altered Metaboanalyst metabolite-gene pathways from the comparison of the tumor region versus the necrotic region. Proteins and lipids used in the joint-pathway analysis were considered up- or downregulated for the specific region (logFC ≥0.58 and logFC ≤-0.58 respectively and with a *p-value* of ≤0.05).

**Upregulated tumor vs. necrose**	**Downregulated tumor vs. necrosis**
Alanine aspartate and glutamate metabolism	alpha-Linolenic acid metabolism
Arginine and proline metabolism	Arachidonic acid metabolism
Arginine biosynthesis	Drug metabolism – other enzymes
Cysteine and methionine metabolism	Glycosylphosphatidylinositol (GPI)-anchor biosynthesis
Glycerolipid metabolism	Linoleic acid metabolism
Glycerophospholipid metabolism	Nicotinate and nicotinamide metabolism
Phenylalanine metabolism	Porphyrin and chlorophyll metabolism
Phenylalanine tyrosine and tryptophan biosynthesis	
Tyrosine metabolism	

Supplementary table 5

Significantly altered proteins and lipids used in the comparison of the tumor region versus the necrotic region. Proteins and lipids were considered up- or downregulated for the specific region (logFC ≥0.58 and logFC ≤-0.58 respectively and with a *p-value* of ≤0.05).

Downregulated proteins tumor vs. necrosis

**Accession number**	**Protein name**
P07357	Complement component C8 alpha chain (Complement component 8 subunit alpha)
P10153	Non-secretory ribonuclease (RNase 2
Q10588	ADP-ribosyl cyclase/cyclic ADP-ribose hydrolase 2 (cADPR hydrolase 2)
Q9H3Z4	DnaJ homolog subfamily C member 5 (CSP)
Q99439	Calponin-2 (Neutral calponin)
P09769	Tyrosine-protein kinase Fgr (Proto-oncogene c-Fgr)
Q15080	Neutrophil cytosol factor 4 (NCF-4)
Q16769	Glutaminyl-peptide cyclotransferase (Glutamyl cyclase)
P09917	Polyunsaturated fatty acid 5-lipoxygenase (Arachidonate 5-lipoxygenase)
P41439	Folate receptor gamma (FR-gamma)
P59665	Neutrophil defensin 1 (HNP-1)
P05164	Myeloperoxidase (MPO)
P08246	Neutrophil elastase (Elastase-2)
P24158	Myeloblastin (AGP7)
P80511	Protein S100-A12 (CAGC)
P02788	Lactotransferrin (Lactoferrin)
P41218	Myeloid cell nuclear differentiation antigen
P08311	Cathepsin G (CG)
P22894	Neutrophil collagenase (PMNL-CL)
P04004	Vitronectin (VN)
P05109	Protein S100-A8 (Calgranulin-A)
P12429	Annexin A3 (Annexin III) (
P12724	Eosinophil cationic protein (ECP)
Q9HD89	Resistin (ADSF)
P06702	Protein S100-A9 (Calgranulin-B)
P80188	Neutrophil gelatinase-associated lipocalin (NGAL)
Q14134	Tripartite motif-containing protein 29
P27105	Stomatin
P08575	Receptor-type tyrosine-protein phosphatase C
P10909	Clusterin (Apolipoprotein J) (Apo-J)
P20160	Azurocidin
P25815	Protein S100-P (S100 calcium-binding protein P)
P28676	Grancalcin
P13671	Complement component C6
O75146	Huntingtin-interacting protein 1-related protein (HIP-12)
P00450	Ceruloplasmin
P20292	Arachidonate 5-lipoxygenase-activating protein (FLAP)
Q14739	Delta(14)-sterol reductase LBR (Delta-14-SR)
P51159	Ras-related protein Rab-27A (Rab-27)
P30536	Translocator protein (PKBS)

P26022	Pentraxin-related protein PTX3
P43652	Afamin (Alpha-Alb)
P26447	Protein S100-A4 (S100 calcium-binding protein A4)
P02748	Complement component C9
P02743	Serum amyloid P-component (SAP)
P01011	Alpha-1-antichymotrypsin (ACT
P00747	Plasminogen
P36222	Chitinase-3-like protein 1) (CGP-39)
P30740	Leukocyte elastase inhibitor (LEI)
P49913	Cathelicidin antimicrobial peptide) (CAP-18
Q6UX06	Olfactomedin-4 (OLM4)
P14780	Matrix metalloproteinase-9 (MMP-9)
P11215	Integrin alpha-M
P02763	Alpha-1-acid glycoprotein 1 (AGP 1)
P00734	Prothrombin
P01033	Metalloproteinase inhibitor 1 (TIMP-1)
P13796	Plastin-2 (L-plastin)
P04083	Annexin A1 (Annexin I)
O00160	Unconventional myosin-If (Myosin-Ie)

Upregulated proteins tumor vs. necrosis

Accession number	Protein name
P35611	Alpha-adducin (Erythrocyte adducin subunit alpha)
P17677	Neuromodulin (Axonal membrane protein GAP-43)
P78324	Tyrosine-protein phosphatase non-receptor type substrate (SHPS-1)
P48681	Nestin
P11137	Microtubule-associated protein 2 (MAP-2)
P00505	Aspartate aminotransferase, mitochondrial (mAspAT)
P14136	Glial fibrillary acidic protein (GFAP)
Q05193	Dynamin-1 (Dynamin I)
Q92777	Synapsin-2 (Synapsin II)
P16949	Stathmin (Leukemia-associated phosphoprotein p18
Q9Y6U3	Scinderin (Adseverin)
Q13442	28 kDa heat- and acid-stable phosphoprotein (PDGF-associated protein)
Q9P2U7	Vesicular glutamate transporter 1 (VGluT1)
Q05682	Caldesmon (CDM)
P78559	Microtubule-associated protein 1A (MAP-1A)
O43301	Heat shock 70 kDa protein 12A
Q96GW7	Brain-enriched hyaluronan-binding protein (BEHAB)
P50993	Sodium/potassium-transporting ATPase subunit alpha-2
P37840	Alpha-synuclein (Non-A beta component of AD amyloid)
Q14894	Ketimine reductase mu-crystallin (NADP-regulated thyroid-hormone-binding protein)
P80723	Brain acid soluble protein 1 (NAP-22)
Q13435	Splicing factor 3B subunit 2 (SAP 145)
P60880	Synaptosomal-associated protein 25 (SNAP-25)
P17600	Synapsin-1 (Brain protein 4.1) (Synapsin I)
P55087	Aquaporin-4 (AQP-4)

P05026	Sodium/potassium-transporting ATPase subunit beta-1
P09471	Guanine nucleotide-binding protein G(o) subunit alpha
P08247	Synaptophysin (Major synaptic vesicle protein p38)
P51674	Neuronal membrane glycoprotein M6-a (M6a)
P21579	Synaptotagmin-1 (Synaptotagmin I) (SytI) (p65)
Q16623	Syntaxin-1A (Neuron-specific antigen HPC-1)
P61764	Syntaxin-binding protein 1 (MUNC18-1)
Q9UQM7	Calcium/calmodulin-dependent protein kinase type II subunit alpha (CaMK-II)
Q16143	Beta-synuclein
P02808	Statherin
P12270	Nucleoprotein TPR
P52306	Rap1 GTPase-GDP dissociation stimulator 1
P60201	Myelin proteolipid protein (PLP) (Lipophilin)
P62993	Growth factor receptor-bound protein 2 (Adapter protein GRB2)
Q9BY11	Protein kinase C and casein kinase substrate in neurons protein 1 (Syndapin-1)
Q9NR46	Endophilin-B2 (SH3 domain-containing GRB2-like protein B2)
Q9Y6R1	Electrogenic sodium bicarbonate cotransporter 1 (kNBC1)

**Downregulated lipids tumor vs. necrosis**	**Upregulated lipids tumor vs. necrosis**
Cer(d18:1/21:0)	PC(31:0)
Galalpha1-4Galbeta-Cer(d18:1_16:0)	PC(32:1)
Galalpha1-4Galbeta-Cer(d18:1_24:1)	PC(34:1)
PC(O-36:2)	PC(34:3)
PC(O-36:2)	PC(38:7)
SM(41:2)	PC(40:7)
SM(42:2)	PC(O-38:4)
TG(50:4)	PE(38:2)
TG(50:5)	PE(38:3)
TG(52:1)	PE(P-16:0/22:6)
TG(52:2)	PS(40:6)
TG(52:4)	SM(36:1)
TG(54:3)	SM(38:1)
TG(54:4)	TG(55:5)
TG(54:6)	TG(56:6)
TG(54:7)	
TG(56:2)	
TG(56:7)	
TG(58:6)	
TG(58:7)	
TG(58:8)	
TG(58:9)	
TG(58:10)	
TG(60:8)	
TG(60:9)	
TG(60:10)	
TG(60:11)	
TG(62:9)	
